# Primer design for quantitative real-time PCR for the emerging Coronavirus SARS-CoV-2

**DOI:** 10.7150/thno.47649

**Published:** 2020-06-01

**Authors:** Dandan Li, Jiawei Zhang, Jinming Li

**Affiliations:** 1 National Center for Clinical Laboratories, Beijing Hospital, National Center of Gerontology; Institute of Geriatric Medicine, Chinese Academy of Medical Sciences, Beijing, People's Republic of China.; 2 Graduate School, Peking Union Medical College, Chinese Academy of Medical Sciences, Beijing 100730, People's Republic of China.; 3 Beijing Engineering Research Center of Laboratory Medicine, Beijing, People's Republic of China.

**Keywords:** coronavirus, SARS-CoV-2, quantitative nucleic acid testing, primer design, sensitivity

## Abstract

In December 2019, a new coronavirus disease (COVID-19) outbreak occurred in Wuhan, China. Severe acute respiratory syndrome-coronavirus-2 (SARS-CoV-2), which is the seventh coronavirus known to infect humans, is highly contagious and has rapidly expanded worldwide since its discovery. Quantitative nucleic acid testing has become the gold standard for diagnosis and guiding clinical decisions regarding the use of antiviral therapy. However, the RT-qPCR assays targeting SARS-CoV-2 have a number of challenges, especially in terms of primer design. Primers are the pivotal components of a RT-qPCR assay. Once virus mutation and recombination occur, it is difficult to effectively diagnose viral infection by existing RT-qPCR primers. Some primers and probes have also been made available on the WHO website for reference. However, no previous review has systematically compared the previously reported primers and probes and described how to design new primers in the event of a new coronavirus infection. This review focuses on how primers and probes can be designed methodically and rationally, and how the sensitivity and specificity of the detection process can be improved. This brief review will be useful for the accurate diagnosis and timely treatment of the new coronavirus pneumonia.

## Introduction

In December 2019, outbreak of a new coronavirus disease (COVID-19), defined by the International Committee on Taxonomy of Viruses, occurred in Wuhan, China. This virus has since been named severe acute respiratory syndrome-coronavirus-2 (SARS-CoV-2) 1. SARS-CoV-2 is highly contagious and has rapidly expanded worldwide since its discovery. As of 12 May, 2020, a total of 4,088,848 cases of SARS-CoV-2 infection have been confirmed worldwide with 283,153 deaths 2, although this may be underestimated due to inadequate testing in many countries 3.

SARS-CoV-2 is an enveloped, positive-sense, single-stranded RNA virus. It possesses a comparatively large genome approaching 30 kb. The genome is arranged in the order of a 5′ untranslated region (UTR)-replicase complex (open reading frame [ORF] 1ab)-structural protein [Spike(S)-Envelope(E)-Membrane (M)-Nucleocapsid (N)]-3′UTR and non-structural ORFs 4. The S-protein has a strong binding affinity to human ACE2 5, and this, along with a long incubation time before manifesting symptoms, means that SARS-CoV-2 has high transmissibility with a mortality rate of approximately 3% 6,7. This also poses considerable challenges regarding the timely treatment and effective prevention and control of COVID-19. To better understand the origin of SARS-CoV-2 and its genetic relationship with other coronaviruses, phylogenetic analyses of coronavirus sequences from various sources can be performed using the neighbour-joining method in MEGA X 8. ORF 1ab, E, and N genes are highly conserved among sarbecoviruses, which are a subgenus of betacoronavirus. The RNA-dependent RNA polymerase (RdRp; nsp12) positioned on ORF 1ab plays a crucial role in RNA synthesis 9,10 with features of rapid mutation and recombination 11. As a result, the conserved regions (ORF 1ab, E, and N genes) are usually selected as the standard target genes for primer and probe design 12.

SARS-CoV-2 has been identified as the seventh coronavirus known to infect humans 13. The previous six coronaviruses were: Human coronavirus (HCoV)-229E, HCoV-NL63, HCoV-HKU1, HCoV-OC43, Middle East respiratory syndrome coronavirus (MERS-CoV), and severe acute respiratory syndrome coronavirus (SARS-CoV). HCoV-229E and HCoV-NL63 are human alphacoronaviruses, whereas HCoV-HKU1, HCoV-OC43, MERS-CoV, and SARS-CoV are human betacoronaviruses. SARS-CoV-2 is a novel betacoronavirus, and is genetically similar to the SARS-like bat coronaviruses in the subgenus Sarbecovirus 14. A complete genomic comparison showed that the new coronavirus has the highest homology with Bat SL-CoVZC45 and Bat SL-CoVZXC21 coronaviruses, but differs from the currently known SARS with a similarity of just 79.7% 15. Although substantial genetic differences exist between the novel coronavirus and other betacoronaviruses, cross-reactions in RT-qPCR or antibody assays for SARS or other betacoronaviruses are possible if the primers and antigenic epitopes are not carefully selected 16. Therefore, it is necessary to design specific primers and probes in the regions with the lowest feasible similarity to other viruses to avoid false detection of SARS-CoV 17.

The timely and accurate laboratory testing of samples from cases under investigation are essential parts of the management of emerging infections. Molecular techniques have been used successfully to identify infectious diseases for many years. As can be observed from the identification path of SARS-CoV-2 18,19, high-throughput sequencing is a powerful tool for the discovery of pathogens, changing the way we respond to infectious disease outbreaks, improving our understanding of disease occurrence, accelerating the identification of pathogens, and promoting data sharing 12,13. However, the analysis method is labour-intensive and time-consuming and, thus, cannot be widely used as a detection method or screening tool.

As sequence information about SARS-CoV-2 has recently become available, quantitative nucleic acid testing has become the gold standard for clinical decisions regarding the use of antiviral therapy 20-22. The most recently developed nucleic acid testing method is quantitative real-time PCR (RT-qPCR) 23,24, although RT-qPCR assay design optimization can be a complicated process. In addition to being rapid, the RT-qPCR assay has the advantage of a standardized protocol that can be easily adapted for the detection of other respiratory viruses. RT-qPCR can be performed under uniform amplification conditions; thus, target-specific primer and probe sets can be utilized 25. Moreover, testing can also be performed using a high-throughput system 26.

However, there are a number of problems related to the RT-qPCR assays targeting SARS-CoV-2. First, the sensitivity of this method is not high in clinical application, and studies have reported cases of positive CT scan results and negative RT-qPCR results at initial presentation 27. Some researchers and clinicians have argued that CT imaging should be used to identify SARS-CoV-2 infection, evaluate treatment response and prognosis of COVID-19 28, 29, but a number of cases have shown progressive multiple peripheral ground-glass opacities in both lungs despite negative RT-qPCR results 30. Furthermore, an accurate detection of SARS-CoV-2 virus can be made from oropharyngeal, nasopharyngeal swabs and/or lower respiratory tract samples (sputum or tracheal aspirates) 31; thus, sampling could also be one of the reasons for low sensitivity. The primers, probes, and reagents used for detection could be another critical factor. Finally, RT-qPCR primers are designed to amplify the target regions of the SARS-CoV-2 genome. However, the novel coronaviruses are RNA viruses, and once mutations and recombination occur, RT-qPCR primers will not be able to effectively amplify the viral sequences. Thus, mutations and recombination can reduce the sensitivity of the RT-qPCR.

Primers are pivotal components of a qPCR assay. Some primers and probes have also been made available on the WHO website for reference. However, no previous review has systematically described how to design primers and probes methodically and rationally in the face of a new coronavirus. Therefore, the focus of the present review is to discuss effective primer designing and improving the sensitivity and specificity of the detection process. This review also discusses the need for accurate diagnosis and timely treatment of the new coronavirus pneumonia.

## Several typical studies regarding the detection process of SARS-CoV-2

**Figure 1:**
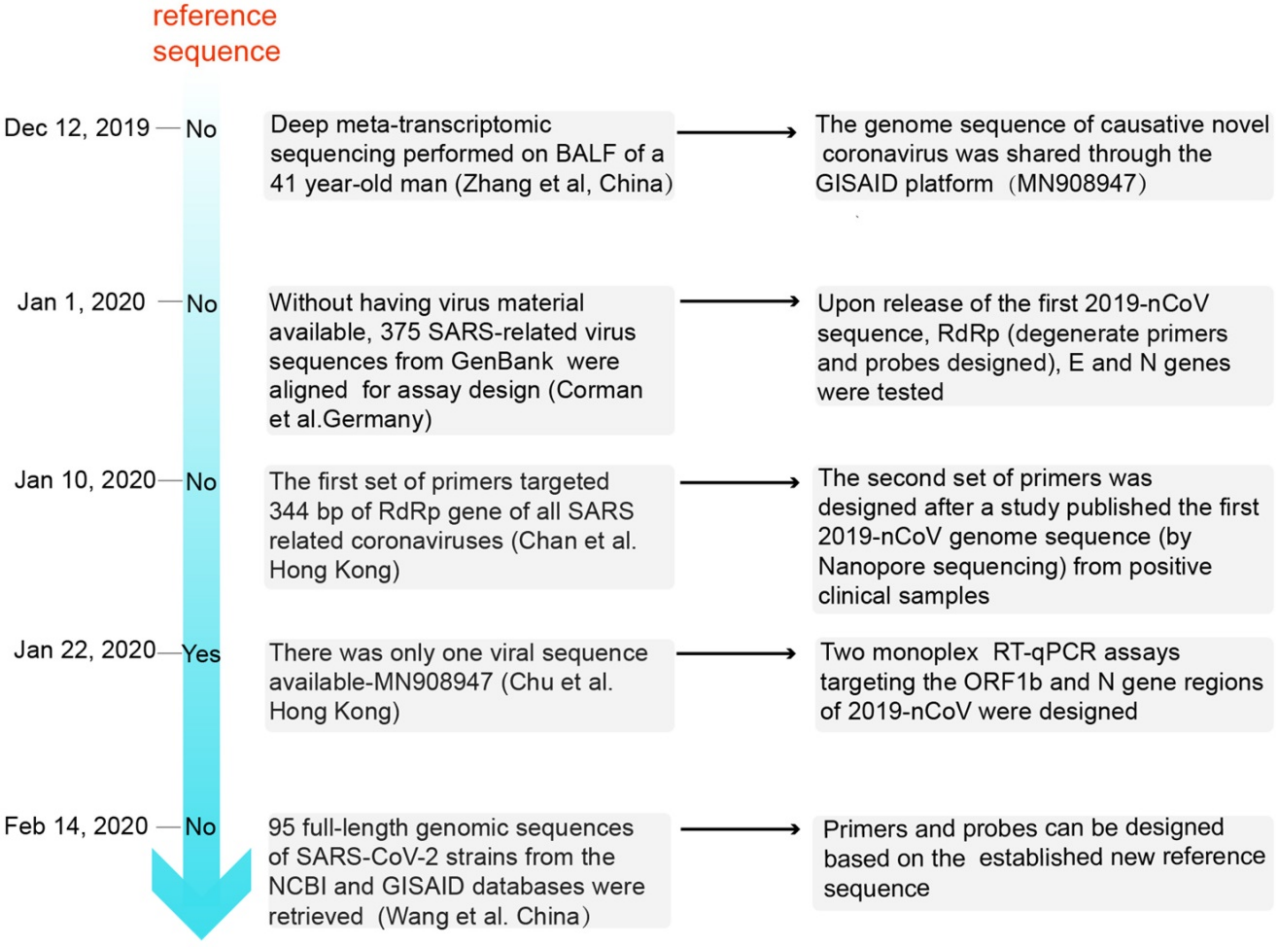
The analysis process of primer and probe design for SARS-CoV-2 in chronological order. BALF: bronchoalveolar lavage fluid; GISAID: Global Initiative on Sharing All Influenza Data.

At present, gene sequences of the six known coronaviruses (HCoV-229E, HCoV-NL63, HCoV-HKU1, HCoV-OC43, MERS-CoV, and SARS-CoV) have been published, and primers and probes can be designed based on the available genomic information. When an unknown coronavirus suddenly emerges and causes a new type of infectious pneumonia, how should we respond? In such a scenario, designing effective primers with high specificity and sensitivity can have immense value for diagnosis, tracking, and tracing. The design of SARS-CoV-2 primers and probes and the step-by-step analysis process to accurately detect the virus are shown in Figure 1.

Zhang et al. were the first to determine the full-length genomic sequence of the SARS-CoV-2 virus 18. On 26 Dec, 2019, six days after the onset of disease, a 41-year-old man with fever, unproductive cough, pain, and weakness was admitted to the Central Hospital of Wuhan. Bronchoalveolar lavage fluid (BALF) was collected to perform deep meta-transcriptomic sequencing. Primers used for RT-qPCR were designed based on the whole genome of WH-Human 1 coronavirus (MN908947). The detailed reference sequences of the viral RNA (GenBank MN908947) are shown in Table 1. The genome sequence of the causative novel coronavirus was shared through the Global Initiative on Sharing All Influenza Data (GISAID) platform (http://www.gisaid.org/) from 12 Jan 2020 32. Subsequently, a number of other SARS-CoV-2 sequences were released; these sequences were found to be almost identical 17.

On 1 Jan 2020, Corman et al. used the close correlation between the SARS-CoV-2 and SARS coronavirus genomes to artificially synthesize the 2019-nCoV sequence and establish an experimental detection process in the absence of virus isolates 33. On 10 Jan 2020, Chan et al reported that two patients with fever, respiratory symptoms, and pulmonary infiltrates on chest radiographs were enrolled at the University of Hong Kong-Shenzhen Hospital. Subsequently, between 11 to 15 Jan 2020, five other members of the patients' family also presented to the hospital for clinical assessment 19. The first set of primers targeted 344 bp of the RdRp gene of all SARS-related coronaviruses. Next, these positive clinical samples were analysed by Nanopore sequencing, and a second set of primers were designed from the SARS-CoV-2 genome sequence and targeted a 158 bp region of the Spike (S) gene of this novel coronavirus 19. Moreover, Won et al. designed primers based on the sequence of the first patient infected with SARS-CoV-2 in Korea 34,35. On 22 Jan 2020, the downloaded sequence and those for SARS coronavirus, bat SARS-like coronaviruses, and other representative coronaviruses among the first publicly available sequences in GenBank (Accession number: MN908947) were edited and aligned. Two sequence regions (ORF1b and N) that are highly conserved among sarbecoviruses were selected for primer and probe design in the study by Chu et al. 14. On 14 Feb 2020, 95 full-length genomic sequences of SARS-CoV-2 strains from the NCBI and GISAID databases were retrieved, and a reference sequence was established. Researchers found that the overall variation in the ORF regions was low; 13 variation sites in the 1a, 1b, S, 3a, M, 8, and N regions were identified, among which positions nt28144 in ORF 8 and nt8782 in ORF 1a showed mutation rates of 30.53% (29/95) and 29.47% (28/95), respectively 36. There may be selective mutations in SARS-CoV-2, and it is necessary to avoid certain regions when designing primers and probes.

Generally, in the face of unknown pathogenic and highly infectious coronaviruses, the first step involves extracting RNA from patient samples and sequencing the whole genome. However, the whole genome sequencing operation is difficult and costly, and it is impossible to use this method for all patients with suspected infection. Genome sequencing can be used as a detection method in a few samples, and the results can be used as a reference sequence to design primers and probes targeting the target gene, so that RT-qPCR detection can be achieved. However, RNA viruses are susceptible to mutations. Shen et al. recently identified a remarkable level of viral diversity in some infected patients, accounting for a median number of four intra-individual viral variants, which is suggestive of the rapid evolution of SARS-CoV-2 37. With the increasing number of cases, results from genome sequencing will also increase. Genome sequences of multiple coronaviruses can be compared to determine the reference sequence. For primer and probe design, it is more reliable and scientifically sound to select conserved regions instead of mutation sites.

## Reference sequence and degenerate primers of SARS-CoV-2

Assays are useful only if the correct target has been identified and used in the assay design. Therefore, it is necessary to know as much information as possible related to the RNA genome sequences. The first step involves obtaining the maximum amount of information from sequence databases; it is crucial to refer to the accession or individual transcript number of related sequences for assay design.

### Necessity for the design of a reference sequence

To design a PCR primer set, a reference sequence is needed to identify the exact sequence being targeted and from where to select the primer pair candidates. Ideally, the reference sequence consists of not only the regions targeted for amplification, but also all the nucleotide sequences that will be involved in the amplification process. This is essential to ensure that the designed primers do not amplify non-specific products 38.

### Establishing the reference sequence

When a new coronavirus is identified, the genome sequence of the virus can first be obtained through standard high-throughput sequencing of patient samples, which can be used as a reference sequence. When the coronavirus spreads further, the sequencing results of different coronavirus strains can be uploaded to the GISAID (https://www.gisaid.org) and NCBI (https://www.ncbi.nlm.nih.gov/) databases. Primer 7.0 can be used to compare the reference nucleotide sequence with those of related human isolates and to analyse the variation at different locations. In addition, the ClustalW program of MEGA (7.0.14) and Mafft (7.450) software can be used to conduct homology analyses and multiple sequence alignments. These sequences can be retrieved from experimental data or online databases. In conclusion, the reference sequence is determined by selecting the highest frequency nucleotide at each position.

### Necessity for designing degenerate primers

Usually, degenerate primers are designed in two different instances. The first is when the target sequence is unknown or presents variability. In such a case, the target sequence can be inferred through the amino acid sequence. Degenerate primers can overcome the uncertainty of the target sequence due to the redundancy of the genetic code 39. The second is when degenerate primers are used to amplify targeted sequences across multiple genotypes by allowing variant primers to simultaneously amplify multiple sequences. Previous studies have detected five different SARS-CoV-2 haplotypes, and the results indicated active genetic recombination 40. The mutation regions may reduce the accuracy of RT-qPCR detection 41. Thus, designing degenerate primers and/or probes is an effective method for addressing the challenge of virus mutations.

### Designing degenerate primers

Primers can be designed as regular or degenerate. Degenerate primer design requires a set of reference sequences. When regular primers are synthesised, only two populations of primers are present in the PCR reaction: the forward and reverse primer populations. When degenerate primers are synthesized, there may be multiple forward and reverse primers. A good principle for designing highly efficient degenerate primers is to establish a limit of degeneracy of up to 64 (the number of different sequences within the mixture primer). It is recommended that the degenerate primers contain R(A/G), Y(C/T), S(C/G), W(A/T), K(G/T), and M(A/C), which have two variants at each position, instead of B(C/G/T), D(A/G/T), H(A/C/T), V(A/C/G), and N(G/A/T/C), which have more than two variants at each position 39.

## Analysis of previously reported primers and probes

Many institutions have studied primer-probe sets for the confirmation of SARS-CoV-2. The molecular diagnosis of COVID-19 is currently carried out by one-step RT-qPCR targeting SARS-CoV-2. Primers and probes have been developed by researchers at China CDC (China), Charité-Universitätsmedizin Berlin Institute of Virology (Germany), The University of Hong Kong (Hong Kong), the National Institute of Health (Thailand), and the Centers for Disease Control and Prevention (CDC, USA) (https://www.who.int/emergencies/diseases/novel-coronavirus-2019/technical-guidance/laboratory-guidance). The primers and probes reported for the SARS-CoV-2 RT-qPCR assays are shown in Table 2.

### Comparison of the target genes: E gene, ORF 1ab, and N gene

**Figure 2:**
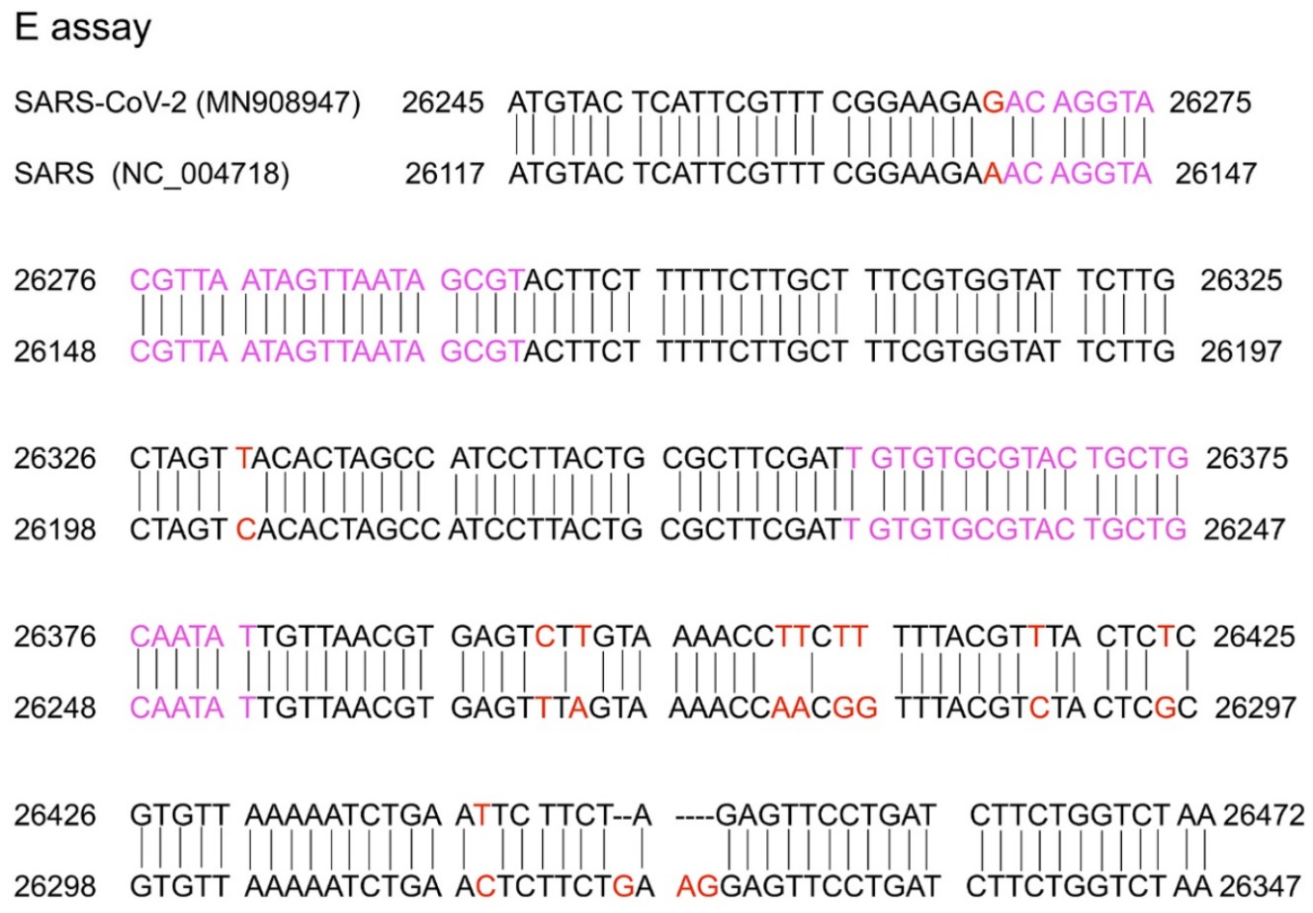
Sequence alignment of the reference (MN908947) and SARS (NC_004718) sequences. Red colour marks the mismatch positions and pink colour marks the forward primer and reverse complementary sequence of the reverse primer in the study by Corman et al. 33.

A conserved region in the structural protein envelope E gene is usually selected for the E gene assay, which normally functions as the first line screening assay. The E genes of the reference (MN908947) and SARS (NC_004718) sequences were aligned using nucleotide BLAST (https://blast.ncbi.nlm.nih.gov/). The results are shown in Figure 2. The E gene primer/probe set described by Corman et al. was found to be more sensitive than those of the N gene and RdRp 47. The position of the primer targeting the E gene is shown in Figure 2 and indicated with pink text. From the alignment of the E gene between the SARS-CoV-2 reference sequence (MN908947) and SARS-CoV (NC_004718), there was 94% homology and the mismatch positions are marked in red in Figure 2. Corman et al. selected the forward and reverse primers in the complete match position so that both SARS-CoV-2 and SARS-CoV could be detected. It is believed that SARS has been eliminated in humans and the last reported human SARS case was detected in 2004 48,49. Samples with positive SARS-CoV results were considered to be infected by SARS-CoV-2 or its related animal coronaviruses. Selecting the common sequence of bat-associated SARS-related viruses is essential to ensure broad sensitivity even in the case of multiple independent acquisitions of variant viruses. Therefore, this pancoronavirus assay is a useful tool for screening sample collections for the presence of all known and potentially unknown coronaviruses. When designing the specific primer/probe targeting the E gene, the mismatch position should be considered (Figure 2).

Compared with the E gene length in SARS-CoV-2, the gene lengths of ORF 1ab and N are longer, and more variation points are present. Therefore, regions in the ORF 1ab and N genes are usually selected for the target-specific forward and reverse primers designed for confirmatory testing. Research has shown that SARS-CoV-2 is distinct from SARS-CoV in the phylogeny of the complete RdRp gene 17. As shown in Table 2, tests for the RdRp gene in Germany and ORF1b-nsp14 gene in Hong Kong used degenerate probes and primers. There are two main reasons for this detection approach. First, the genetic diversity of 2019-nCoV in humans and animals is yet to be fully determined. Second, many laboratories lack positive controls for SARS-CoV-2. The serially diluted RNA samples extracted from SARS-CoV-infected cells usually work as the positive control (obtained via the European virus archive global (EVAg), https://www.european-virus-archive.com) 50. SARS-CoV-2 isolation is not recommended as a routine diagnostic procedure. In vitro transcribed RNA of the target gene of SARS-CoV-2 can be cloned into a plasmid as the RNA standard. On one hand, the degenerate probes and primers can detect the positive control (SARS-CoV), and on the other hand, they can detect SARS-CoV-2. For example, Corman et al. formulated a broad-range probe reacting with SARS-CoV and SARS-CoV-2, an additional specific probe that reacts only with SARS-CoV-2. In their study, RdRp and E genes are more sensitive than N genes, and the RdRp gene assay is recommended as a confirmatory assay. However, Chu et al. found that the N gene assay was approximately 10 times more sensitive than the ORF-1b gene assay in detecting positive clinical specimens from two patients via RT-qPCR 14; based on their detection performance, the N gene RT-qPCR is recommended as a screening assay, and the ORF1b-nsp14 assay is recommended as a confirmatory assay. Further experimental studies are required to evaluate the sensitivity of the primer/probe set shown in Table 2. For one target gene, there may exist many regions for which the primer/probe is designed. We believe that it is more reasonable to compare the primer/probe sensitivity for the same target gene among different test regions. However, if the regions of multiple target genes are different in each study, the comparability among different studies is lost. Alternatively, the primer/probe sets from different studies can be evaluated under the same experimental conditions with the same test samples.

The USA CDC designed N1, N2, and N3 genes (shown in Table 2) as the target of the SARS-CoV-2 Real-Time RT-PCR Diagnostic Panel. In the SARS-CoV-2 RT-PCR assay, N1 and N2 were designed for the specific detection of SARS-CoV-2, whereas N3 was designed for the universal detection of SARS-like coronaviruses 51. Experiments found that the CDC N1 and N2 primer/probe sets performed better than the N3 set, and the N3 primer and probe set was removed from the Diagnostic Panel of revision 2 for the CDC 2019-nCoV Real-Time RT-PCR Diagnostic Panel.

Researchers in Korea performed experiments to compare the previously reported primer-probe sets from the WHO for molecular diagnosis. Their results revealed that in the case of targeting the RdRp/ORF1 region, the ORF 1ab (China) set might be more sensitive than others. 2019-nCoV_N2 (USA) and NIID_2019-nCOV_N (Japan) sets may be recommended for the sensitive RT-qPCR assay of the N region. Therefore, an appropriate combination of the ORF 1ab (China), 2019-nCoV_N2 (USA), and NIID_2019-nCOV_N (Japan) sets should be selected for sensitive and reliable laboratory confirmation of SARS-CoV-2 52. Other researchers found that the E gene primer/probe set described by Corman et al. and the N2 set developed by the CDC were the most sensitive assays after evaluating seven different primer/probe sets (RdRp, E, and N genes in the study by Corman et al. and N1, N2, and N3 by CDC and RdRp developed by the University of Washington Clinical Virology Lab) 47.

### Improving the sensitivity of the RT-qPCR assay

From the perspective of primers, there are three main ways to improve the sensitivity of RT-qPCR: primer concentrations, degenerate primers, and multi-target detection. First, primer concentrations for probe-based assays were 300-900 nM 53. The primer concentrations of all tests (Table 2) were within this range (data not shown), and increasing the concentration of primers appropriately may improve the sensitivity of RT-qPCR. For example, Vijgen et al. found that primer concentrations increased up to 400 nm per reaction can improve the sensitivity of the assay 54. Second, degenerate primers are used to cope with the genetic diversity of SARS-CoV-2. Finally, regarding multi-target detection, screening assays with a single target region are more vulnerable to sequence variations than dual- or triple-target assays 55,56. Overall, the E gene assay was used for pan-sarbecovirus detection, and the RdRp assay and the N gene assay may eventually be utilised as confirmatory assays.

In addition to sensitivity, specificity is an important indicator when considering primers and probes. Whether there is cross-reactivity is an important aspect of evaluating specificity. The proportion of co-infections has been underestimated due to either a lack of sensitivity or the limitations of virus detection technologies 57. As co-infections can occur, all patients that meet the suspected case definition should be tested for COVID-19 regardless of whether another respiratory pathogen is found. However, all tests shown in Table 2 indicate no cross-reactivity with HCoV-229E, HCoV-NL63, HCoV-HKU1, HCoV-OC43, MERS-CoV, and other respiratory viruses, indicating that the specificity of the primers and probes listed in Table 2 is high.

## General principles of primer/probe design strategy

A successful PCR reaction depends on a number of factors, most of which are related to the primer design quality. The primer length should be between 16 and 28 nucleotides, as shown in Table 2. Primer melting temperatures (Tm) should be between 50°C and 62°C, depending on the GC content. The GC content of the primer is preferably 45-55%. Moreover, the GC content and Tm value of the upstream and downstream primers should be kept close. The Tm difference between the two primers should be lower than 5°C 58, and the annealing temperature should be 5°C lower than that for the primer with the lowest Tm. Because the target sequence of the primers is variable for degenerate primers, a good approach is to set annealing temperatures between 50°C and 55°C, although specific primers could be used to estimate the PCR conditions more accurately. For the polymorphisms in the SARS-CoV-2 genomes, there may be mismatches in primers or probes that cause false-negative conditions 59. The significance of the regular examination of primers and probes has been emphasized 60. False negative results can be reduced by designing primers and probes with melting temperatures >60°C and run conditions of the assay with annealing temperatures at 55°C to tolerate one to two mismatches when amplifying a coronavirus gene with higher variability.

The 5′- and 3′-ends of the primers play different roles; although the 5′-end contributes marginally, matching the 3′ end is critical for PCR efficiency amplification. It has been found that a single mismatch in the last three nucleotides counting from the 3′-end is acceptable, but the second impaired position drops efficiency below the detectable level on agarose gel electrophoresis 61.The base at the terminal 3' bases of the primer generally does not require A nucleotide. More attention should be paid to the last five bases; in particular, no more than three G/C residues should be included in the last five bases and the primer should never end with three consecutive G/C residues, such as GGG or CCC, otherwise the complementary, dimer, or hairpin structure at the 3′ end of the primer may cause the PCR reaction to fail. Multiple pairs of primers in the same amplification system can also increase the probability of primer dimer formation, which can be predicted by related software such as Oligo7 57. The bases of primers should be randomly distributed, and it is suggested that there should be no more than four complementary bases between the forward and reverse primers. As reported in previous studies, primer dimers can be prevented by modifying with 2′-O-methyl bases in the penultimate base 26. The closer the probe is to the upstream primer, the higher the hydrolysis efficiency. However, it should be kept above 3 bp. The 5' end of the probe cannot start with a G base because it can quench the fluorophore 62. As shown in Table 2, most of the probes do not start with a G base except for that for the N gene developed in Hong Kong.

**Figure 3:**
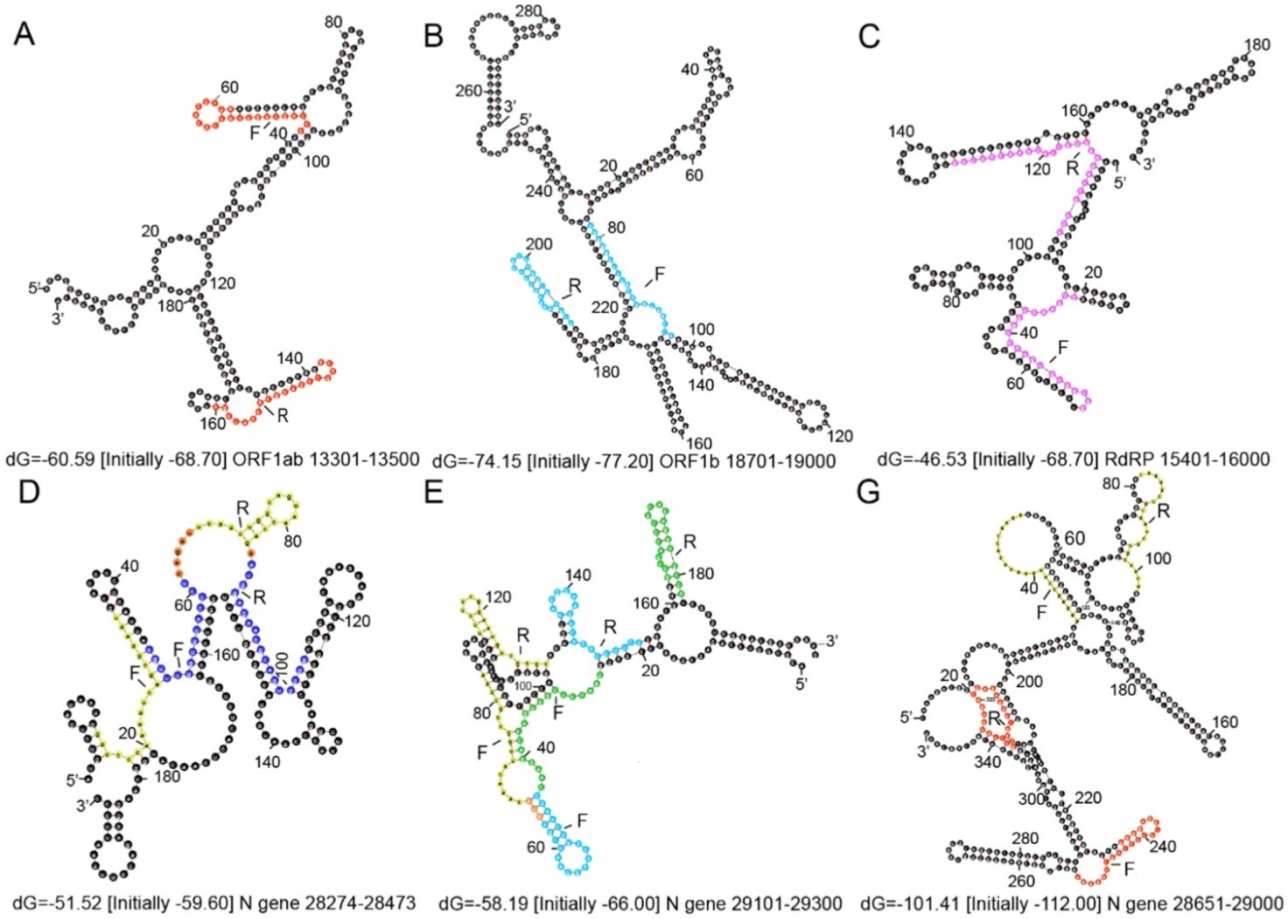
** Secondary structures of the ORF 1ab and N gene fragments amplified using the forward and reverse primers. (A)** The forward primer and reverse complementary sequence of the reverse primer for ORF 1ab from China are indicated in red. **(B)** The forward primer and reverse complementary sequence of the reverse primer for ORF1b from Hong Kong are shown in light blue. **(C)** The forward primer and reverse complementary sequence of the reverse primer for RdRp from Germany are shown in pink. **(D)** The forward primer and reverse complementary sequence of the reverse primer for the N1 gene from USA are shown in yellow and the N gene in Thailand are shown in dark blue. **(E)** The forward primer and reverse complementary sequence of the reverse primer for the N2 gene from USA are shown in yellow, the N gene from Hong Kong are shown in light blue, and the N gene from Japan are shown in green. **(G)** The forward primer and reverse complementary sequence of the reverse primer for the N3 gene from USA are shown in yellow and the N gene from China in red. Orange represents the overlap. F represents the forward primer and R represents the reverse complementary sequence of the reverse primer.

After designing the primer/probe following the general principle strategy, in silico analysis is important for specificity or exclusivity testing. For example, an alignment was performed with the oligonucleotide primer and probe sequences of the CDC 2019 nCoV RT-qPCR diagnostic panel with all publicly available nucleic acid sequences for 2019-nCoV in GenBank. NCBI Primer-BLAST can be used to confirm primer specificity. It is best to confirm specificity again after primer probe design is completed, especially since the primers and probes of multiple RT-qPCR need to be mixed together. It has also been suggested that primer/probe sets are selected from conserved sequences on stem structures to avoid loop structures 63, which may be different from the general principle that recommends designing primers to avoid secondary structure in templates. As a general principle, this is correct because the template can be paired to form the stem by itself, which may not be conducive to accurate PCR quantification. However, what we want to emphasize in this review is that, firstly, SARS-CoV-2 is an RNA virus, which is unstable and easily degraded by RNase A 64. The stem structure is more stable than the loops that are easily cleaved 65,66 and by avoiding loops and targeting stems, the original RNA value can be nearly reached, even if the SARS-CoV-2 RNA is degraded under improper transport and storage conditions before analysis, resulting in broader applicability 63. In addition, during reverse transcription in the RT-qPCR assay, the temperature is mostly set to 55°C, and this temperature is conducive to the effective opening of RNA secondary structures 67. During the amplification process, through denaturation, the secondary structures will also be opened. It is important to check that all secondary structures have a Tm (°C) less than the qPCR annealing temperature (normally 55-60°C) to ensure effective amplification 68. Therefore, we suggest that in the primer design for SARS-CoV-2, it is more effective to design the primer targeting position on the stem, at least to make sure that the 3 ' terminal bases are on the stem (as shown in Figure 3). However, further experiments are needed to explore and verify this.

The secondary structures of the ORF 1ab and N gene fragments amplified using the forward and reverse primers from Table 2 are shown in Figure 3. The secondary structures of the target genes were predicted by Mfold (http://mfold.rna.albany.edu/?qZmfold) using the minimum free energy method. The forward and reverse primers designed by each country avoided the loop positions as much as possible. Researchers also revealed that if the loop size, determined by the distance between paired palindrome elements, is less than 11 base pairs and the stem length, determined by the alignment of the palindrome elements, exceeds 14 hydrogen bonds, then there may be stem loop interference in the primer binding site 69. According to this, there are no stem loop interferences observed in the binding sites of primers developed in each country (Figure 3).

**Figure 4:**
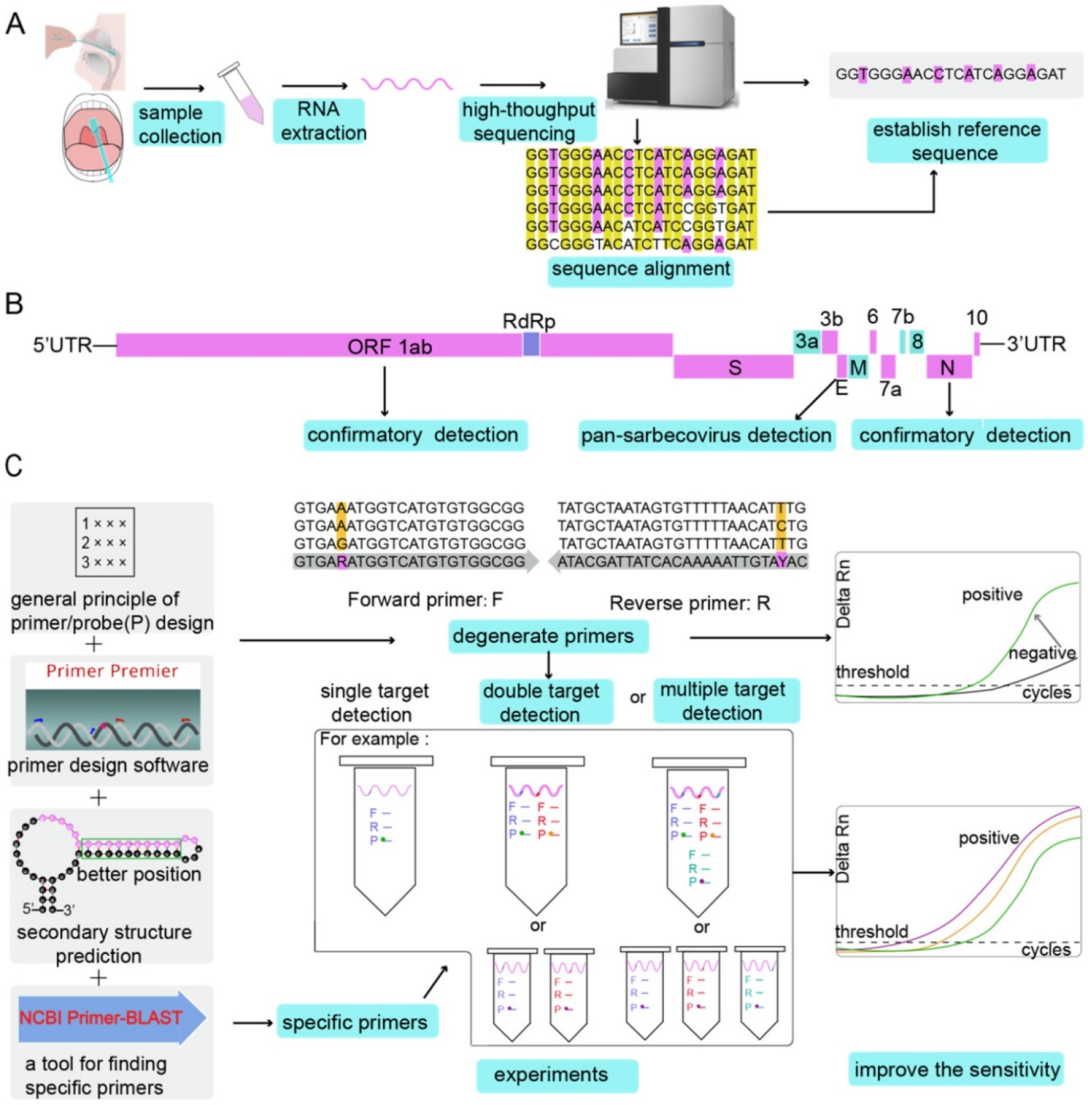
** The entire flow of primer design. (A)** Establishment of the reference sequence. Different types of samples are collected, RNA is extracted, and then high-throughput sequencing is performed to determine the reference sequence. Alternatively, after multiple sequencing results are aligned, the reference sequence can be determined. **(B)** Identification of the target genes. By comparing the SARS-CoV-2 genome with other bat-associated SARS-related viral genomes, conserved and non-conserved regions were identified. Conserved regions such as ORF 1ab, E gene and N gene can be used as target genes for designing primers. Primers are designed at the same target region (E gene) of SARS-CoV-2 genome and bat-associated SARS-related viral genes for pan-sarbecovirus detection. Specific primer probes can be designed for specific target gene regions of SARS-CoV-2 (ORF 1ab and N gene) for confirmation experiments. **(C)** Improving the sensitivity of the RT-qPCR assay. Primers are designed according to the general principles of primers or by using software. Degenerate primers can increase the sensitivity of the reaction. When using primers to detect the target gene, if the target gene is mutated, the universal primer may amplify, but the degenerate primer can make a false negative result into a positive result (Grey heads indicate that false negative results become positive results). After considering the secondary structure of the target gene, the target gene on the stem can prevent RNA from being cleaved by RNase A, which can also increase the detection rate. NCBI Primer-BLAST verifies the specificity of the primers. In the experimental process of the designed primer probe, dual-target detection and multi-target detection can also improve the sensitivity of the reaction. The probe is labelled with different fluorescent dyes, or each target gene is detected in a separate assay with the probes labelled with the same fluorescent dye.

The primer and probe sequences can also be designed using software tools 70,71. The commonly used primer design tools are listed in Table 3. Even if the same tools are used, the same suggested primers and probes may not always be the same, but this does not indicate that the quality of primer design is poor. For example, the primers and probes designed for the ORF 1ab of SARS-CoV-2 have different sequences distributed by the Chinese CDC and the USA CDC. Indeed, there are many primer and probe combinations that can meet the requirements of RT-qPCR experiments, so the highest quality combination does not always need to be used. Regardless of how advanced the algorithm of the design tool is, the performance of the probe primer ultimately depends on the experimental data and whether there are non-specific products to be confirmed by gel electrophoresis after PCR or a dissociation curve (which is recommended). The amplification conditions also need to be continuously optimized through experiments. For example, the optimal Tm and optimized oligo concentrations were eventually confirmed by temperature gradient experiments. The parameters given by the design tool are for reference only and should never replace the subsequent confirmation process. From an economic point of view, it is generally recommended to design a probe and three corresponding pairs of primers for a target and determine the best primer combination after experimental screening. The entire flow of the primer design is shown in Figure 4.

It is clear that no diagnostic modality is perfect. In order to obtain RT-qPCR results with high accuracy, each step must be performed satisfactorily in the preanalytical, analytical, and post-analytical steps. It is important to avoid sample contamination and ensure adequate procedures for specimen collection, handling, transport, and storage. For the analytical procedures, many problems, such as active viral recombination, a lack of harmonization of primers and probes, and non-specific PCR annealing, should be addressed. Finally, the healthcare personnel must be educated on the interpretation of RT-qPCR results. Despite high sensitivity, negative RT-qPCR results observed at one or two time points are insufficient to exclude SARS-CoV-2 infection in patients, especially for those with typical clinical presentations or clear epidemic indications. Therefore, negative results do not preclude SARS-CoV-2 infection and should not be used as the sole basis for treatment or other patient management decisions. Negative results must be combined with clinical observations, patient history, and epidemiological information.

Each RT-qPCR run should include both external and internal controls, and laboratories are encouraged to participate in external quality assessment schemes that should be established as soon as possible to monitor analytical quality and harmonize the assays. In many studies 34,44, the internal control primer set targeted the human ribonuclease P (RNase P) protein. This protein is an ubiquitous enzyme in all cells and cellular compartments 72. The GAPDH gene can also be used as the target gene for the internal control 17,57. In order to identify regions of the viral genome, further analysis of the molecular targets is needed to achieve the highest possible diagnostic accuracy. The primer/probe set should be evaluated with further experiments, so that more sensitive and specific primers/probes can be developed. Ordering primers and probes to perform validation testing on functionality and potential contaminants is also recommended 73.

## Conclusion

As RT-qPCR is the current gold standard for the etiological diagnosis of SARS-CoV-2 infection, the diagnostic accuracy of this technique should be a foremost prerequisite. This review provides an overview of the most crucial components of a RT-qPCR assay-primer design (summarised in Figure 4). First, it is important to identify the reference sequence using a step-by-step process, and eventually align the sequence, if required. The gene targets include the N, E, ORF 1ab (or RdRP), and S genes. The more conserved E gene is the target for the pan-coronavirus assay, while N and RdRP genes mainly function as targets for confirmatory assays. After considering the basic rules of primer/probe design, and degenerate primers/probes against SARS-CoV can be used as a positive control; moreover, primers that account for the variation in coronavirus sequences can be developed. The single-target method is prone to lead to false negative results once a site in the target sequence is mutated. Thus, dual-target or multi-target detection can be additionally used to improve detection sensitivity. After multiple pairs of primers are designed, regardless of whether they are designed manually or by software, further verification is necessary. In silico analysis of primer and probe sequences is needed to analyse the specificity. However, the experimental conformation is more important. Only by evaluating the diagnostic ability of the designed primers and probes on a number of samples, we can identify primers/probes with good sensitivity and specificity. Furthermore, the reaction conditions of the assay, such as the concentration of the primers and probes, Tm, and annealing temperature, must be optimized for maximum accuracy of the results.

## Figures and Tables

**Table 1:** Detailed information of the reference sequence MN908947

ORF	Function	Location (nt)	Length (bp)
5' UTR		1-265	265
1ab		266-21,555	21,290
1a	Encoded nonstructural proteins (nsp1 to nsp11), essential for viral replication, viral assembly, immune response modulation, etc.	--	--
1b	Encoded nonstructural proteins (nsp12 to nsp16), essential for viral replication	--	--
S	Binding to cell receptor and mediating virus-cell fusion	21,563-25,384	3,822
3a	Accessory protein	25,393-26,220	828
3b	Accessory protein	25,765-26,220	456
E	Envelope protein, virus assembly, and morphogenesis	26,245-26,472	228
M	Membrane protein, virus assembly	26,523-27,191	669
6	Accessory protein	27,202-27,387	186
7a	Accessory protein	27,394-27,759	366
7b	Accessory protein	27,756-27,887	132
8	Accessory protein	27,894-28,259	366
N	Nucleocapsid protein, forms complexes with genomic RNA, interaction with M protein for viral assembly	28,274-29,533	1,260
10	Accessory protein	29,558-29,674	117
3' UTR		29,675-29,903	229

**Table 2:** Information regarding the primers and probes reported for SARS-CoV-2 real-time reverse-transcription PCR assays

Target genes	Country	Name	Sequence (5' → 3')	Reference sequence	Nucleotide position	Reference
RdRp	China	Forward primer: Reverse primer:	CAAGTGGGGTAAGGCTAGACTTT ACTTAGGATAATCCCAACCCAT	--	14961-14983 15283-15304	19
RdRp /nCoV IP2	Paris	Forward primer: Reverse primer: Probe	ATGAGCTTAGTCCTGTTG CTCCCTTTGTTGTGTTGT Hex-AGATGTCTTGTGCTGCCGGTA-BHQ-1	SARS-CoV, NC_004718	12621-12727	42
RdRp/nCoV IP4	Paris	Forward primer: Reverse primer: Probe	GGTAACTGGTATGATTTCG CTGGTCAAGGTTAATATAGG FAM-TCATACAAACCACGCCAGG-BHQ-1	SARS-CoV, NC_004718	14010-14116	42
RdRp gene	Germany	Forward primer; Probe 2; Probe 1; Reverse primer	GTGARATGGTCATGTGTGGCGG FAM-CAGGTGGAACCTCATCAGGAGATGC-BBQ FAM-CCAGGTGGWACRTCATCMGGTGATGC-BBQ CARATGTTAAASACACTATTAGCATA (W=A/T; R=A/G; M=A/C; S=G/C)	MN908947	15431-15452 15470-15494 15469-15494 15505-15530	33
ORF1a	China	Forward primer: Reverse primer: Probe	AGAAGATTGGTTAGATGATGATAGT TTCCATCTCTAATTGAGGTTGAACC FAM-TCCTCACTGCCGTCTTGTTGACCA-BHQ1	Alignment of sequenced virus genomes	--	17
ORF 1ab	China	Forward primer: Reverse primer: Probe	TGATGATACTCTCTGACGATGCTGT CTCAGTCCAACATTTTGCTTCAGA ROX-ATGCATCTCAAGGTCTAGTG-MGB	MN908947	15704-15728 15823-15846 15749-15768	18
ORF 1ab	China	Forward primer: Reverse primer: Probe	CCCTGTGGGTTTTACACTTAA ACGATTGTGCATCAGCTGA FAM-CCGTCTGCGGTATGTGGAAAGGTTATGG-BHQ	MN908947	13342-13362 13442-13460 13377-13404	43
ORF1b-nsp14	Hong Kong	Forward primer: Reverse primer: Probe	TGGGGYTTTACRGGTAACCT AACRCGCTTAACAAAGCACTC FAM/ZEN-TAGTTGTGATGCWATCATGACTAG-IBFQ (W=A/T; Y=C/T; R=A/G)	MN908947	18778-18797 18889-18909 18849-18872	14
N gene	China	Forward primer: Reverse primer: Probe	GGGGAACTTCTCCTGCTAGAAT CAGACATTTTGCTCTCAAGCTG FAM-TTGCTGCTGCTTGACAGATT-TAMRA	MN908947	28881-28902 28958-28979 28934-28953	43
N gene	Hong Kong	Forward primer, Reverse primer, Probe	TAATCAGACAAGGAACTGATTA CGAAGGTGTGACTTCCATG FAM/ZEN-GCAAATTGTGCAATTTGCGG-IBFQ	MN908947	29145-29166 29236-29254 29179-29198	14
N1 gene	USA	Forward primer: Reverse primer: Probe	GAC CCC AAA ATCAGCGAA AT TCTGGTTACTGCCAGTTGAATCTG FAM-ACCCCGCATTACGTTTGGTGGACC-BHQ1	MN908947	28287-28306 28335-28358 28309-28332	44
N2 gene	USA	Forward primer: Reverse primer: Probe	TTACAA ACATTGGCCGCA AA GCGCGACATTCCGAAGAA FAM-ACA ATTTGCCCCCAGCGTTAG-BHQ1	MN908947	29164-29183 29213-29230 29188-29210	44
N3 gene	USA	Forward primer: Reverse primer: Probe	GGGAGCCTTGAA TAC ACC AAA A TGTAGCACG ATTGCAGCATTG FAM-AYCACATTGGCACCCGCA ATCCTG-BHQ1	MN908947	28681-28702 28732-28752 28704-28727	44
NIID_2019-nCOV_N_F2	Japan	Forward primer: Reverse primer: Probe	AAATTTTGGGGACCAGGAAC TGGCAGCTGTGTAGGTCAAC FAM-ATGTCGCGCATTGGCATGGA-BHQ1	MN908947	29125-29144 29263-29282 29222-29241	45
N gene	Thailand	Forward primer: Reverse primer: Probe	CGTTTGGTGGACCCTCAGAT CCCCACTGCGTTCTCCATT FAM-CAACTGGCAGTAACCA-BQH1	MN908947	28320-28339 28358-28376 28341-28356	46
E gene	Germany	Forward primer: Reverse primer: Probe	ACAGGTACGTTAATAGTTAATAGCGT ATATTGCAGCAGTACGCACACA FAM-ACACTAGCCATCCTTACTGCGCTTCG-BBQ	MN908947	26269-26294 26360-26381 26332-26357	33
Spike	China	Forward primer: Reverse primer:	CCTACTAAATTAAATGATCTCTGCTTTACT CAAGCTATAACGCAGCCTGTA	MN938384 MN975262	22712-22741 22849-22869	19
RNase P gene (RP)	USA	Forward primer: Reverse primer: Probe	AGATTTGGACCTGCGAGCG GAGCGGCTGTCTCCACAAGT FAM -TTCTGACCTGAAGGCTCTGCGCG-BHQ-1	CDC internal control	--	44
GAPDH	China	Forward primer: Reverse primer: Probe	TCAAGAAGGTGGTGAAGCAGG CAGCGTCAAAGGTGGAGGAGT VIC-CCTCAAGGGCATCCTGGGCTACACT-BHQ1	internal control	--	17

**Abbreviations:** E, envelope protein gene; M, membrane protein gene; N, nucleocapsid protein gene; ORF, open reading frame; RdRp, RNA-dependent RNA polymerase gene; FAM, 6-carboxyfluorescein; BBQ, blackberry quencher; BHQ-1, black hole quencher 1; TAMRA, carboxytetramethylrhodamine; IBFQ, Iowa Black FQ.

**Table 3:** List of practical primer design tools

Aim	Name	Software or Website
Secondary structure prediction	Mfold	http://mfold.rna.albany.edu/?qZmfold
	RNAfold	http://rna.tbi.univie.ac.at//cgi-bin/RNAWebSuite/RNAfold.cgi
	RNAStructure	http://e.informer.com/rna.urmc.rochester.edu/RNAstructure.html
	SFold	http://sfold.wadsworth.org/cgi-bin/index.pl
Primer design	Primer Premier	Software
	Primer Express	Software
	DNAMAN	Software
	Oligo 7	Software
	Prime3 Plus	http://primer3plus.com/cgi-bin/dev/primer3plus.cgi
	QuantPrime	https://www.quantprime.de/
	Primer Blast	https://www.ncbi.nlm.nih.gov/tools/primer-blast/index.cgi
	Primer bank	https://pga.mgh.harvard.edu/primerbank/
	JCVI Primer Designer	https://sourceforge.net/projects/primerdesigner/
Test the primer specificity	NCBI Primer-BLAST	https://www.ncbi.nlm.nih.gov/tools/primer-blast/
	Primer Blast	https://www.ncbi.nlm.nih.gov/tools/primer-blast/index.cgi

## Abbreviations

SARS-CoV-2: severe acute respiratory syndrome coronavirus 2
MERS-CoV: Middle East respiratory syndrome coronavirus
SARS-CoV: severe acute respiratory syndrome coronavirus
ORF: Open reading frame
S: Spike
E: Envelope
M: Membrane
N: Nucleocapsid
RT-qPCR: Quantitative real-time PCR
RdRp: RNA-dependent RNA polymerase
BALF: Bronchoalveolar lavage fluid
GISAID: Global Initiative on Sharing All Influenza Data
FAM: 6-carboxyfluorescein
BBQ: Blackberry quencher
BHQ-1: Black hole quencher 1
TAMRA: Carboxytetramethylrhodamine
IBFQ: Iowa Black FQ
CDC: Centers for Disease Control and Prevention
EVAg: European virus archive global
